# A Review of Nonlinear Filtering Algorithms in Integrated Navigation Systems

**DOI:** 10.3390/s25206462

**Published:** 2025-10-19

**Authors:** Jiaqian Si, Yanxiong Niu, Botao Wang

**Affiliations:** 1 School of Instrumentation and Optoelectronic Engineering, Beihang University, Beijing 100191, China; sijiaqianbuaa@163.com (J.S.); niuyx@buaa.edu.cn (Y.N.); 2 School of Electronic and Electrical Engineering, Shangqiu Normal University, Shangqiu 476000, China

**Keywords:** nonlinear filtering, integrated navigation, Kalman filtering

## Abstract

Nonlinear filtering algorithms have significant implications in the optimal estimation of navigation states and in improving the accuracy, reliability, and robustness of navigation systems. This manuscript surveys the developments of the nonlinear filtering algorithms (extended Kalman filtering (EKF), unscented Kalman filtering (UKF), Cubature Kalman filtering (CKF), particle filtering (PF), neural network filtering (NNF)) and adaptive/robust KF in integrated navigation systems. The principle, application, and existing problems of these nonlinear filtering algorithms are mainly studied, and the comparative analysis and prospect are carried out.

## 1. Introduction

With the continuous development of navigation and positioning technology, especially in the aerospace field, there are higher requirements for the accuracy of navigation and positioning [1]. Global Navigation Satellite System (GNSS) systems can provide high-precision navigation functions for satellites, vehicles, ships, and military fields worldwide, and are widely used in military and civilian fields. However, changes in the external environment have a significant impact on the accuracy of GNSS, especially in remote areas such as forests and mountainous regions where navigation satellite signals are weak [2]. An Inertial Navigation System (INS) is an autonomous navigation technology that relies entirely on internal sensors, does not require external positioning signals or communication support, and is not affected by electromagnetic interference or external radiation. The characteristic of an inertial navigation system is that the signal has high positioning accuracy in a short period of time [3,4]. However, due to the limitations of the manufacturing level, the inertial navigation equipment will produce certain initial errors, and long-term integration accumulation will cause excessive errors and reduce system stability. Therefore, although inertial navigation has high accuracy and independence in the short term, it is not suitable for long-term navigation due to error accumulation issues. GNSS can only provide position and speed, not direction information. In addition, the low data rate of GNSS may affect the positioning accuracy [5,6,7].

It is difficult for GNSS, INS, and celestial navigation systems (CNS) to meet navigation performance requirements when used alone. An effective way to improve the overall performance of navigation systems is to adopt combined navigation technology [8,9,10,11,12]. The integrated navigation system significantly improves the positioning accuracy, system reliability, and environmental adaptability through multi-source information fusion, while effectively overcoming the limitations of a single system [13,14,15,16,17,18].

The integrated navigation of GNSS and an inertial navigation system is essentially a multi-sensor information fusion system, and the data fusion technology involved is a major factor in determining whether the navigation system can meet the real-time requirements of engineering applications [19,20,21,22]. In the integrated navigation of GNSS/INS, the data fusion filtering algorithms used include the Kalman filtering algorithm and its improved algorithms, such as extended Kalman filtering, unscented Kalman filtering, adaptive Kalman filtering algorithm, etc. [23,24,25,26].

The standard Kalman filter is an algorithm that uses the optimal estimation of the system state through the equation of state of a linear system [27]. Obtaining the optimal estimation of physical parameters by processing a series of actual measurement data with errors. The Kalman filter can recursively update the state estimation, enabling the navigation system to adapt to environmental changes in real time and provide accurate position and velocity information. The standard Kalman filter is linear, but the signal typically contains nonlinear components [28,29,30]. Therefore, with the rapid development of navigation systems both domestically and internationally, the state estimation values obtained by Kalman filters are difficult to meet the higher accuracy requirements of navigation positioning. Due to the complexity of noise and measurement, as well as dynamic changes in the system, Kalman filtering is relatively weak in dealing with uncertain interference in complex environments.

In practical engineering applications, considering that most integrated navigation systems are nonlinear, traditional Kalman filtering can only solve the estimation problem of Gaussian noise linear systems [31,32]. For the integrated navigation of GNS/INS, if a linear model is still used to describe the integrated navigation system, the sensors (such as gyroscopes and accelerometers) in the integrated navigation system will produce errors over time, which will gradually accumulate and lead to a decrease in navigation accuracy. When the accuracy of inertial devices is poor or the carrier undergoes large maneuvering movements, the misalignment angle of the inertial navigation system will be large, and the nonlinear factors in the INS/GPS integrated navigation system cannot be ignored. The traditional linear small error equation cannot match the actual system. Continuing to use a traditional linear Kalman filter to filter the linear small error model of the system will lead to the divergence of the filtering state [33,34,35].

In order to address nonlinear problems, researchers have conducted extensive research on optimal estimation methods, and a large number of nonlinear filtering algorithms have emerged. This paper reviews the research progress of nonlinear filtering methods represented by extended Kalman filtering (EKF), unscented Kalman filtering (UKF), Cubature Kalman filtering (CKF), particle filtering (PF), neural network filtering (NNF), and Adaptive/Robust KF in integrated navigation systems in recent years. Systematically analyzed and compared the development trends of these nonlinear filtering methods in integrated navigation systems, providing a reference for the application and development of integrated navigation systems.

## 2. Kalman Filtering (KF)

The Kalman filter is an algorithm based on the equations of a linear system. A Kalman filter can optimally estimate the state of the system by observing the input and output data of the system. This algorithm not only involves filtering, but also includes the prediction of future data. Kalman filtering aims to provide an efficient and computationally inexpensive method to estimate process states and minimize estimation errors. The equations describing the dynamic characteristics of the system are as follows:

(1) $$X_{k+1}=AX_{k}+B\mu_{k}+\omega_{k}$$

(2) $$Z_{k}=HX_{k}+v_{k}$$

X is the estimated state of the system at a certain time $t_{k}$. *A* is the transition matrix of the system from time $t_{k}$ to $t_{k+1}$. B is the control matrix, which describes the influence of external control on the state. $\mu_{k}$ is the control input at time k. $\omega_{k}$ is process noise or interference. $Z_{k}$ is the measurement vector at time k. H is the observation matrix at time k. $v_{k}$ is the noise vector at time k.

Kalman filtering is divided into two steps: a prediction step and a correction step. The calculation process of these two steps is as follows:

(3) $${\overset{\wedge }{X}}_{k+1\left|k\right.}=A{\overset{\wedge }{X}}_{k\left|k\right.}+B\mu_{k}$$

(4) $$P_{k+1\left|k\right.}=AP_{k\left|k\right.}A^{T}+Q_{k}$$

(5) $$P_{k+1\left|k+1\right.}=(1-K_{k+1}H)P_{k+1\left|k\right.}$$

(6) $$K_{k+1}=P_{k+1\left|k\right.}H^{T}{\left(HP_{k+1\left|k\right.}H^{T}+R_{k+1}\right)}^{-1}$$

(7) $${\overset{\wedge }{X}}_{k+1\left|k+1\right.}={\overset{\wedge }{X}}_{k+1\left|k\right.}+K_{k+1}(Z_{k+1}-H{\overset{\wedge }{X}}_{k+1\left|k\right.})$$

where ${\overset{\wedge }{X}}_{k+1\left|k\right.}$ is the predicted system state vector, the superscript ∧ denotes the prior estimate. ${\overset{\wedge }{X}}_{k+1\left|k\right.}$ is the predicted state vector of the system at time step k. ${\overset{\wedge }{X}}_{k+1\left|k+1\right.}$ is the estimated state vector of the system in time step k+1. $P_{k\left|k\right.}$ is the estimated uncertainty (covariance) matrix of the current state. $P_{k+1\left|k\right.}$ is the prediction estimation covariance matrix of the next state. k+1 is the Kalman filter gain at the time step k+1. The covariance matrix can also be described as follows:

(8) $$e_{k+1}=X_{k+1}-{\overset{\wedge }{X}}_{k+1\left|k+1\right.}$$

(9) $$P_{k+1\left|k+1\right.}=E(e_{k+1}e_{k+1}^{T})=E((X_{k+1}-{\overset{\wedge }{X}}_{k+1\left|k+1\right.})\times {\left(X_{k+1}-{\overset{\wedge }{X}}_{k+1\left|k+1\right.}\right)}^{T})$$

The covariance matrix reflects the expected value and the observed value. The smaller the covariance matrix, the more accurate the estimated state is. The Kalman filtering method is an efficient recursive estimation algorithm. Its core principle is to dynamically modify the prediction of the target state by combining the state estimation of the previous time with the measurement value of the current time. However, the Kalman filter is based on the assumption of a linear system, that is, the state transition equation and measurement equation of the system are linear. In practical applications, many systems are nonlinear. In order to make the Kalman filter suitable for nonlinear systems, EKF, UKF, CKF, and other algorithms are proposed.

## 3. Nonlinear Filtering Algorithms

### 3.1. Extended Kalman Filtering (EKF)

The core of nonlinear filtering is the approximate estimation method, which linearizes and approximates nonlinear functions through mathematical transformations and then converts nonlinear models into linear models. This type of method is also known as the function linearization approximation-based method, which commonly uses Taylor polynomial expansion for function linearization approximation. The earliest, most widely used, and representative method in this category is the EKF algorithm. The first-order linearization result of the Taylor polynomial expansion of EKF is used to linearize and approximate the nonlinear function, and then the KF algorithm is applied to filter and solve the linearized system [36]. The nonlinear system space expressed in states is as follows:

(10) $$X_{k}=f(X_{k-1},u_{k})+w_{k}$$

(11) $$Y_{k}=h(X_{k})+v_{k^{\prime }}$$

where *f*(x) and *h*(x) are nonlinear functions. EKF adopts Taylor expansion at the mean value, and is usually approximated by a first-order Taylor expansion. The formula is as follows:

(12) $$f(x)=f(x_{0})+f^{\prime }(x_{0})(x-x_{0})+o(x-x_{0})$$

(13) $$\begin{array}{l} F_{k}={\left.\frac{\partial f}{\partial x}\right|}_{{\overset{\wedge }{x}}_{k-1\left|k-1\right.}}=\frac{f(x_{k-1})-f({\overset{\wedge }{x}}_{k-1\left|k-1\right.})}{x_{k-1}-{\overset{\wedge }{x}}_{k-1\left|k-1\right.}}{\overset{\wedge }{x}}_{k\left|k-1\right.}\\ =f({\overset{\wedge }{x}}_{k-1\left|k-1\right.})+F_{k}(x_{k-1}-{\overset{\wedge }{x}}_{k-1\left|k-1\right.})+w_{k} \end{array}$$

The steps of predicting the EKF and the update steps are described as follows:

(14) $${\overset{\wedge }{x}}_{k\left|k-1\right.}=f({\overset{\wedge }{x}}_{k-1\left|k-1\right.},u_{k})$$

(15) $$P_{k\left|k-1\right.}=F_{k}P_{k-1\left|k-1\right.}F_{k}^{T}+Q_{k}$$

(16) $${\overset{\wedge }{x}}_{k\left|k\right.}={\overset{\wedge }{x}}_{k\left|k-1\right.}+K_{k}(Z_{k}-h_{k}({\overset{\wedge }{x}}_{k\left|k-1\right.}))$$

(17) $$K_{k}=P_{k\left|k-1\right.}H_{k}^{T}{\left(H_{k}P_{k\left|k-1\right.}H_{k}^{T}+R_{k}\right)}^{-1}$$

(18) $$P_{k\left|k\right.}=(1-K_{k}H_{k})P_{k\left|k-1\right.}$$

where *F_k_* and *H_k_* are the Jacobian matrices of functions *f*(x) and *H*(x), respectively. By using Taylor expansion, EKF can be applied to nonlinear systems.

INS and GNS have been used in modern urban road transportation systems to provide navigation and positioning information for people. However, due to the complexity of urban roads and the diversity of underground locations, it is difficult to accurately describe the measurement noise covariance of the integrated navigation system, and the measurement noise covariance will directly affect the stability and positioning accuracy of the system. Therefore, domestic and foreign researchers have conducted relevant studies on this.

Chen (2021) [36] et al. proposed an adaptive extended Kalman filter algorithm to reduce the impact of noise covariance uncertainty on GNSS signal errors caused by various complex scenarios in urban environments and the inability to accurately model the measurement noise covariance matrix during the extended Kalman filter process, which undermines the performance of the extended Kalman filter algorithm. This algorithm enables the states and actions in the extended Kalman filter algorithm to autonomously select the noise covariance matrix based on the process noise variance. Simultaneously, design a pruning algorithm to remove the inappropriate selection of the noise covariance matrix and improve performance. Simulation and field test results show that the positioning accuracy of the adaptive extended Kalman filter algorithm is superior to that of the traditional extended Kalman filter algorithm.

Yin (2023) [37] et al. proposed a robust adaptive extended Kalman filter algorithm for GNSS/INS integrated navigation. Obtain the covariance matrix of dynamic measurement noise by analyzing the position accuracy factor, measurement factor, and position standard deviation. When the measurement is affected, an adaptive filtering algorithm is used to suppress outliers. The experimental results show that the proposed adaptive extended Kalman filter algorithm has better anti-interference and stability, and can effectively reduce the impact of external abnormal interference on the GNSS/INS integrated navigation results. Compared with the classic EKF, adaptive Kalman filtering algorithm, and robust Kalman filtering algorithm, this method has improved the positioning accuracy by 72.43%, 2.54%, and 47.82%, respectively.

The process noise characteristics directly affect the optimal performance of the Kalman filter, resulting in the inability to provide the required estimate when there is non-Gaussian mean noise in the system. An H-infinity filter is a robust filtering method suitable for systems with uncertainty and noise. Unlike Kalman filtering, the H-infinity filter achieves state estimation by optimizing the robustness of the system, making it particularly suitable for complex and uncertain environments. However, achieving a balance between robustness and estimation accuracy is a challenge. Yazdkhasti (2023) [38] et al. proposed an adaptive H-infinity filter extended Kalman filter (AHEKF) to address this issue. This type of filter has high robustness and accuracy, and is controlled by adaptive algorithms to mitigate the effects of time-varying noise characteristics and abnormal data.

In order to achieve high accuracy, the algorithm is applied to the fusion of two independent sensor data, while reducing the impact of time-varying noise characteristics, high initial uncertainty, and abnormal data on the state estimation accuracy of the integrated navigation system. The results show that the accuracy and robustness of AHEKF are more than 50% higher than those of the Standard H-infinity filter, and the convergence speed of the estimation error is more than 2.5 times faster.

In addition, in the INS/GNSS integrated navigation system, various obstacles and signal interference during signal transmission can cause unstable signals received by the satellite receiver. In addition, under non-Gaussian noise, the performance of traditional Kalman filters will significantly decrease. To address this issue, a Maximum Versoria Criterion Extended Kalman Filter (MVC-EKF) algorithm has been proposed, which solves the robustness problem of traditional EKF under non-Gaussian noise conditions compared to traditional EKF. Among all kinds of non-Gaussian noise, the MVC has the advantages of a small amount of exponential calculation and a small steady-state error. The opening of the versoria function is steep, the bottom is flat, and no exponential operation is required. Therefore, the Kalman filter can be improved by using the versoria function instead of the Gaussian kernel function. In response to abnormal measurement situations, this algorithm assigns smaller weight values to them, thereby greatly reducing the impact of abnormal measurements on the state estimation of the integrated navigation system. Through simulation of the SINS/GNSS integrated navigation system, the performance comparison between MVCEKF and other Kalman filtering algorithms (including EKF and Maximum Correlation entry Criteria (MCC)-EKF) was demonstrated. The results indicate that the proposed MVC-EKF algorithm exhibits stronger robustness than other Kalman filtering algorithms in non-stationary measurement processes [39].

The calculation process of the EKF algorithm is relatively efficient, and it can update the system status in real-time at each time step. This makes EKF very suitable for integrated navigation systems requiring rapid response and real-time processing, such as unmanned aerial vehicles, autonomous vehicles, etc.

Although EKF has been widely used in nonlinear filtering of integrated navigation systems, it still has theoretical limitations. When the system nonlinearity is severe, ignoring the high-order terms of the Taylor expansion will cause an increase in linearization error, leading to an increase in filtering error or even divergence of the EKF. Moreover, the calculation of the Jacobian matrix is complex and computationally intensive, making it difficult to implement in practical applications, and sometimes even difficult to obtain the Jacobian matrix of nonlinear functions. Therefore, the EKF algorithm is only applicable to weakly nonlinear systems and requires a certain level of computational complexity. In summary, due to the unavoidable truncation error caused by function approximation, the estimation accuracy and applicability of methods based on linear function approximation are limited.

### 3.2. Unscented Kalman Filtering (UKF)

Due to the low accuracy of the extended Kalman filter algorithm in computing nonlinear systems, it is only suitable for handling systems with relatively weak nonlinearity. For the highly nonlinear system of integrated navigation, it is difficult to achieve ideal filtering results using the extended Kalman filter algorithm. In this regard, it is necessary to find a filtering method that is superior to the extended Kalman filter algorithm to handle systems with strong nonlinearity. Julier (1997) [39] et al. proposed an unscented Kalman filter algorithm based on the unscented transformation, which approximates the probability distribution of nonlinear systems instead of directly approximating nonlinear functions like the extended Kalman filter algorithm. UKF has higher nonlinear approximation accuracy than EKF and does not require the calculation of the Jacobian matrix. Therefore, this algorithm performs better in practical engineering applications.

UKF generates a set of sigma points to simulate the state transition relationship of nonlinear systems. The process of generating the sigma point set $\xi_{k-1}$ is as follows:

(19) $$\mu =\sum_{i=0}^{2n}w_{i}x_{k+1\left|k\right.}(i)$$

(20) $$\sum =\sum_{i=0}^{2n}w_{i}(x_{i}-\mu_{x}){\left(x_{i}-u_{x}\right)}^{T}$$

(21) $$X_{0}=\mu$$

(22) $$w_{0}=\frac{\lambda }{n+\lambda }$$

(23) $$X_{i}=\mu +{\left(\sqrt{(n+\lambda )\sum }\right)}_{i}(i=1,2,\ldots ,n)$$

(24) $$w_{i}=\frac{1}{2(n+\lambda )}(i=1,2,\ldots ,n)$$

(25) $$X_{i}=\mu -{\left(\sqrt{(n+\lambda )\sum }\right)}_{i-n}(i=n+1,n+2,\ldots ,2n)$$

(26) $$w_{i}=\frac{1}{2(n+\lambda )}(i=n+1,n+2,\ldots ,2n)$$

Compared with EKF, UKF is suitable for systems with strong nonlinearity. Al Bitar (2021) [10] et al. proposed a new system that combines an unscented Kalman filter with a nonlinear autoregressive neural network with external inputs to improve the position and velocity accuracy of the inertial navigation system integrated navigation during the downtime of the GNSS. A module based on a nonlinear autoregressive neural network is used to predict the measurement updates of the extended Kalman filter algorithm during the downtime of GNSS. Simultaneously, design a new offline method for selecting the optimal input for nonlinear autoregressive neural networks and conduct testing. The performance of the proposed system was experimentally verified using a real dataset, and the results showed that the method of using a nonlinear autoregressive neural network-assisted extended Kalman filter algorithm was superior to other methods using different neural network input configurations.

**Figure 1:**
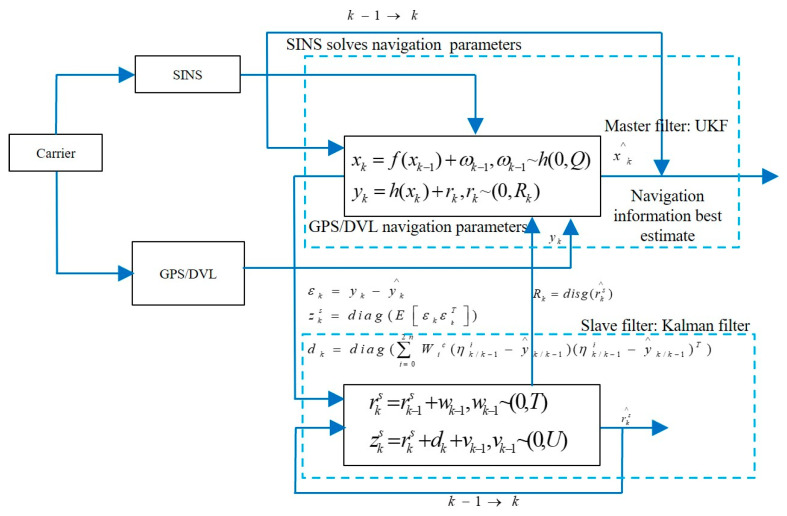
Integrated navigation algorithm based on dual adaptive unscented Kalman filter.

In order to solve the problem of linear or nonlinear integrated navigation systems with time-varying measurement noise covariance, Lyu (2024) [14] et al. developed a nested dual Kalman filter framework structure, as shown in Figure 1. The system consists of UKF and EKF, and is based on a nonlinear UKF to achieve state estimation of the integrated navigation system. The proposed dual adaptive UKF (dual AUKF) algorithm has good anti-interference performance and measurement accuracy in response to various interferences during the measurement process, and has been verified through airborne and shipborne navigation experiments.

**Figure 2:**
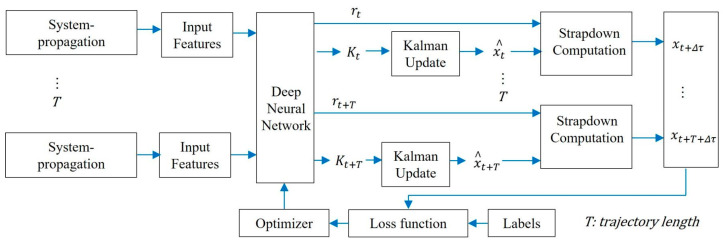
ES-EKF system architecture combined with deep learning.

The performance of traditional EKF is affected by system noise models, model errors, and other factors. To overcome these limitations, Li (2024) [40] et al. proposed applying deep learning algorithms to error-state EKF (ES-EKF), a hybrid algorithm that can improve the accuracy of GNSS/INS integrated navigation systems. As shown in Figure 2, a CNN-LSTM (Long Short Term Memory, LSTM) neural network is used to learn the inherent characteristics of the GNSS/INS integrated system and IMU measurement values, in order to provide estimated Kalman gain and IMU error. The measurement results of accelerometers and gyroscopes were simulated using different error models to evaluate the proposed algorithm, and the results were compared with traditional ES-EKF. The experimental results indicate significant improvements in position, velocity, and heading estimation using the proposed algorithm. CNN-LSTM is capable of learning accumulated IMU errors in strapdown computation and adjusting IMU error estimates to compensate for low-frequency IMU measurements. It also overcomes the shortcomings of the system model, as well as unknown processes and measurement noise.

The INS/GNSS/CNS integrated navigation system is an ideal navigation method for hypersonic vehicles. However, due to the susceptibility of GNSS and CNS measurements to interference during highly dynamic maneuvers, this integration is difficult to achieve optimal navigation solutions using existing information fusion technologies. A multi-sensor information dispersion fusion method based on robust UKF for INS/GNSS/CNS combination is proposed to address the above issues [41]. Firstly, a robust UKF local state estimation method based on fault detection was established. Evaluate abnormal measurement values of GNSS and CNS based on the Mahalanobis distance. Then, determine the scalar factor and introduce it into the innovation covariance to reduce the Kalman gain and improve the robustness of UKF to outlier measurements. Introducing a determined scalar factor into the covariance to reduce the Kalman gain is beneficial for improving the robustness of the UKF system. In addition, multi-sensor fusion technology is introduced in nonlinear systems to fuse local state estimation of INS/GNSS and INS/CNS integrated systems using the Unscented transform within the framework of minimum variance estimation. This method can obtain globally optimal fusion estimation results for abnormal measurements of INS/GNSS/CNS combined hypersonic aircraft navigation systems. Through physical simulation and comparative analysis, it has been proven that the proposed method has excellent performance.

**Figure 3:**
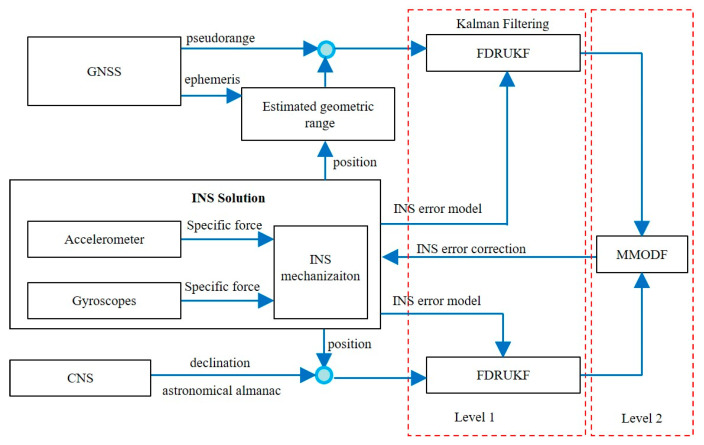
System architecture diagram of INS/GNSS/CNS integration.

As shown in Figure 3, the designed INS/GNSS/CNS integrated system features a two-level information fusion architecture. At the first level, the proposed robust UKF algorithm based on fault detection tightly couples INS with GNSS and CNS, handling the interference from abnormal measurements in parallel for state estimation. At the second level, local state estimation is depicted in Figure 3. An integrated system architecture for INS/GNSS/CNS has been designed. The state information from the INS/GNSS and INS/CNS subsystems is fused using a multi-sensor optimal data fusion method to obtain a global state estimate.

The state vector dimension of multi-source integrated navigation systems is generally much higher than the measurement vector dimension. The standard UKF algorithm is only applicable to systems with low dimensionality of state variables. When the dimensionality of variables exceeds a certain range, the application of the UKF algorithm to system state equations or measurement equations with linear features will generate a large number of redundant calculations. In this case, the computational cost of UT transformation is much greater than that of matrix operations in KF, greatly reducing the real-time performance of the system. In addition, the standard UKF algorithm is only applicable to multi-source integrated navigation systems with prior statistical information of known, accurate system process models and measurement models. For actual systems in non-ideal environments in engineering, it is difficult to establish an accurate system model due to uncertain factors such as sudden changes in system parameters, instantaneous interference, unknown system noise statistics, and unknown drift, resulting in a decrease in estimation accuracy and even divergence of the UKF algorithm.

### 3.3. Cubature Kalman Filter (CKF)

The volumetric Kalman filter was first proposed by Canadian scholars Arasaratnam (2009) [42] et al. The volumetric Kalman filter algorithm has a strong ability to solve the state estimation of nonlinear systems. For strongly nonlinear systems, the volumetric Kalman filter algorithm overcomes the limitations of the unscented Kalman filter algorithm and the extended Kalman filter algorithm, and has better stability. The CKF algorithm is based on the third-order spherical radial volume criterion, using a set of volume points to approximate the state mean and covariance of nonlinear systems with additive Gaussian white noise. The procedure for generating the sigma point set $x_{k}$ is as follows:

(27) $$\begin{array}{l} x_{i}=\sqrt{\frac{m}{2}}\left\{\left.\left[\begin{array}{c} 1\\ 0\\ \vdots \\ 0 \end{array}\right],\ldots ,\left[\begin{array}{c} 1\\ 0\\ \vdots \\ 1 \end{array}\right],\left[\begin{array}{c} -1\\ 0\\ \vdots \\ 0 \end{array}\right],\ldots ,\left[\begin{array}{c} 1\\ 0\\ \vdots \\ 0 \end{array}\right]\right\}\right.\\ w_{i}=\frac{1}{m};i=1,2\ldots ,m\\ m=2n \end{array}$$

where *n* is the dimension of the state vector and *m* is the number of cubature points. The cubature points set contains 2*n* volume points, which are used to construct the n-dimensional state vector. All points in the cubature point set have only positive weights and are located in a region. Like UKF, each cubature point set will get a new point set after transforming the nonlinear function. UKF largely depends on parameters, while CKF does not need parameters. In addition, by using the Cubature transformation, CKF can deal with third-order nonlinear factors.

Gao (2017) [43] et al. conducted a comparative study on information fusion using EKF, UKF, and CKF based on the tight combination model of the Beidou inertial navigation system. The experimental results showed that the volumetric Kalman filter algorithm outperformed the extended Kalman filter algorithm and the unscented Kalman filter algorithm in terms of accuracy. Chang (2021) [44] et al. proposed a new fuzzy strong tracking volume Kalman filter data fusion algorithm to further improve the positioning accuracy and stability of GNSS/INS integrated navigation. Design a fuzzy logic controller for the strong tracking volume Kalman filter, aiming to enhance the filter’s ability to recognize and respond to dynamics. Conduct separate tests on the vectors to reveal the dynamic characteristics within the velocity and position states. Perform parallel fuzzy inference to generate a time-varying smoothing factor matrix, which helps the strong tracking volume Kalman filter obtain multiple fading factors distributed to each state variable. Through simulation analysis and experimental verification, the algorithm has good robustness and high accuracy.

In order to address the issues of unknown measurement noise covariance and measurement outliers in INS/GNSS integrated navigation systems, Zhong (2023) [45] et al. proposed a CKF based on variational Bayesian adaptive particle filtering. This algorithm utilizes the Bayesian method to estimate measurement noise and introduces the minimum mean square error criterion to improve the robustness of the system. And the estimation accuracy and robustness of the proposed algorithm were verified through drone testing.

In addition, Liu (2022) [46] et al. proposed a variational Bayesian Kalman filter to address the issue of poor measurement accuracy in integrated navigation systems. By estimating the measurement noise using the variational Bayesian method and introducing the maximum correlation criterion, the robustness of the filter is provided. The proposed filter has been validated for its effectiveness through numerical simulations and experiments.

**Figure 4:**
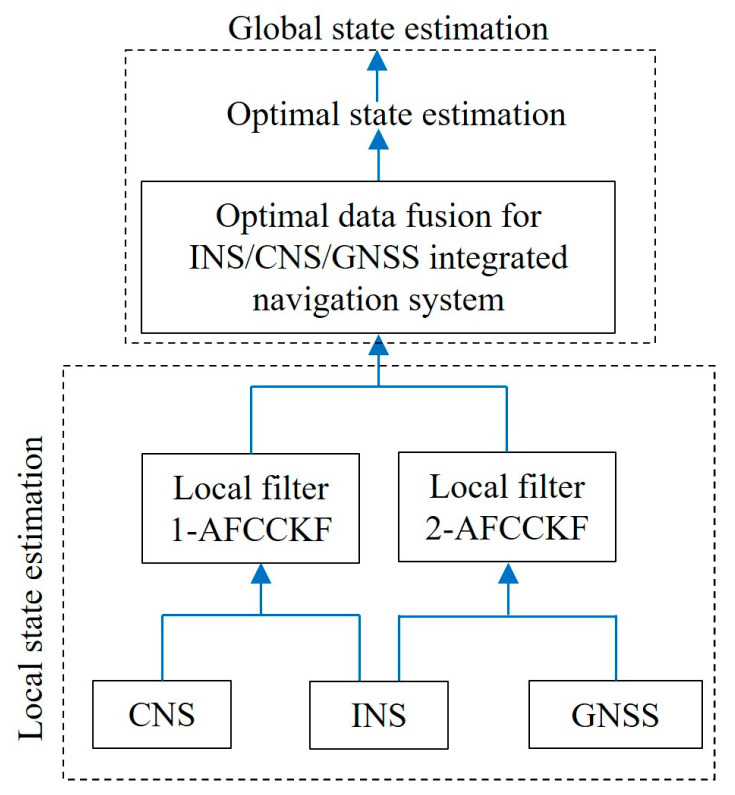
The basic framework of algorithms.

Aiming at the influence of non-Gaussian noise in nonlinear systems, Meng (2022) [47] et al. proposed a Cauchy kernel Cubature Kalman filter to. The strong robustness of the filter under non-Gaussian interference was verified through nonlinear system simulation. Liu (2022) [48] et al. proposed an optimal data fusion algorithm for high-order CKF to address the performance degradation caused by non-Gaussian noise and process modeling errors in integrated navigation systems. As shown in Figure 4, in global state estimation, high-order quadrature criteria are used to fuse local state estimation and obtain the optimal state estimation. The experimental results show that the algorithm significantly improves the robustness of the integrated navigation system in non-Gaussian noise.

**Figure 5:**
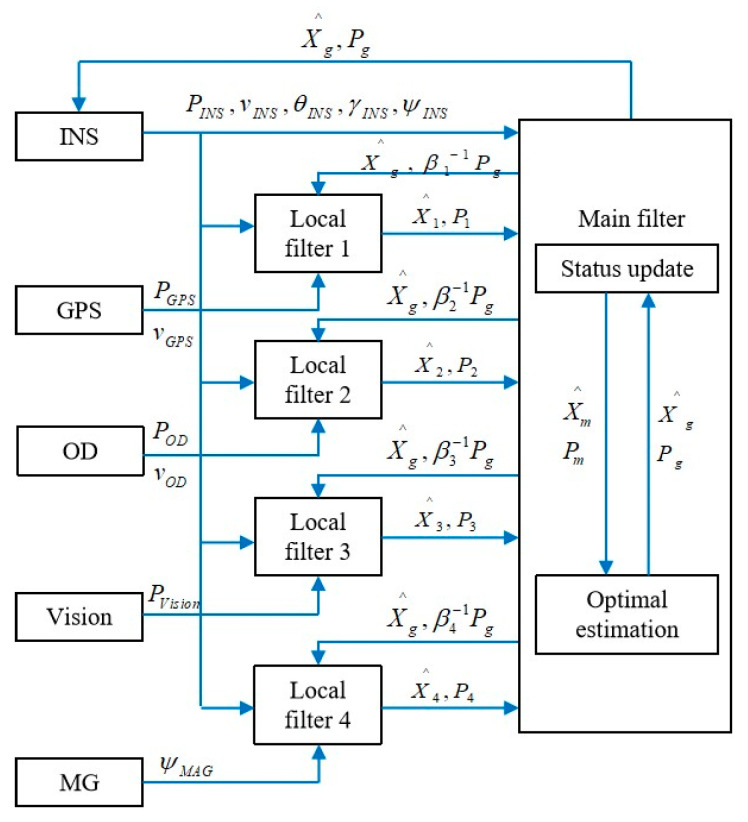
Schematic diagram of a multi-source fusion navigation system.

Zhu (2023) [49] et al. proposed an INS/GPS/odometer/vision/magnetometer fusion navigation solution to address the issue of GPS interference in complex environments for unmanned ground vehicles, while considering the requirements of low cost, high accuracy, and high reliability of navigation systems. As shown in Figure 5. This algorithm has good robustness and convergence speed, improving its positioning ability in GPS interference environments. The proposed multi-source fusion navigation algorithm has good robustness and convergence speed, which improves its positioning ability in a GPS jamming environment.

In order to improve the convergence speed and robustness of nonlinear filters in GNS/INS integrated navigation systems, a CKF based on improved robust estimation is proposed. Through multiple iterations, the convergence speed is improved, and the error compensation effect is improved. In addition, the robustness of the filter is improved by introducing the Geman-McClure loss function. The field experimental results show that the proposed algorithm has better performance in compensating navigation errors when the signal is interrupted [50].

The traditional Volume Kalman Filter (CKF) is a good filter for the combination of Inertial Navigation System (INS) and Global Positioning System (GPS) under Gaussian noise. However, when INS/GPS systems are subjected to complex non-Gaussian interference, CKF may provide significantly biased estimates. Dang (2022) [51] et al. proposed a robust nonlinear Kalman filter-minimum error entry with inertial points (MEEF-CKF) to address this issue. The MEEF-CKF algorithm involves several main steps, including regression model construction, robust state estimation, and free parameter optimization. More specifically, in the first step, consider the residuals caused by linearized nonlinear functions to construct the regression model. Within the framework of a regression model, a MEEF-CKF model was established by solving an optimization problem based on minimum error entropy and reference point (MEEF). In MEEF-CKF, a new optimization method is proposed to adaptively determine the free parameters. In addition, the computational complexity and convergence of the MEEF-CKF algorithm were analyzed, demonstrating its computational complexity and convergence characteristics. The robustness of the MEEF-CKF algorithm was verified through Monte Carlo simulation of INS/GPS combined target tracking under complex non-Gaussian noise.

Wang (2022) [52] et al. proposed an improved maximum correntropy cubature Kalman filter (MCCKF) that utilizes a resampling-free sigma-point update framework (SUF) The proposed algorithm, named RMCCKF, enhances its robustness under non-Gaussian noise and improves the efficiency of measurement updates. The effectiveness of the algorithm was verified through numerical simulations and on-site experiments of GNSS/INS in vehicles. In the on-board GNSS/INS integrated navigation system, RMCCKF not only improves positioning accuracy compared to MCCKF, but also reduces heading error from 1.77° to 0.26°.

In summary, researchers have continuously optimized the algorithm structure of CKF to improve the convergence speed and estimation accuracy of filters, in response to the nonlinearity and complexity of integrated navigation systems. In addition, to address potential issues such as model uncertainty and noise interference in practical applications, researchers have also introduced advanced technologies such as adaptive filtering and robust filtering to enhance the robustness and stability of CKF. In high-dimensional systems, the computational complexity of CKF significantly increases, making it difficult to ensure real-time performance and accuracy. In addition, the nonlinear characteristics of high-dimensional systems are more complex, and the selection and weighting of volume rules also face challenges. In practical applications, the model of integrated navigation systems often has uncertainties, such as sensor errors, environmental interference, etc. These uncertain factors may lead to a mismatch between the model and the actual system, thereby affecting the filtering effect. CKF has certain limitations in dealing with model uncertainty, making it difficult to accurately estimate the true state of the system.

### 3.4. Particle Filter (PF)

The particle filter algorithm originates from the Monte Carlo idea and can use known data to predict future data. In theory, it can handle all nonlinear and non-Gaussian systems. The specific steps of particle filtering are as follows:

(1) Initialize particle sets $S_{i}^{N}$. Give each particle the same weight w=1/N.

(2) Predict the posterior probability density of the system and sample the particle set $S_{t}^{N}$ at time t from the state transition distribution $p(s_{t}\left|s_{t-1},u_{i}\right.)$.

(28) $$S_{t}^{N}∼p(s_{t}\left|s_{t-1},u_{i}\right.)$$

(3) According to the observed value $z_{t}$, the process of modifying the estimated posterior probability can be divided into two steps: importance sampling and resampling.

- Importance sampling

The weight is calculated as follows:

(29) $$\begin{array}{l} w_{t}^{i}=\frac{p(s_{t}^{i}\left|z_{1:t},u_{1:t}\right.)}{p(s_{t}^{i}\left|z_{1:t-1},u_{1:t}\right.)}\\ =\frac{p(z_{t}\left|s_{t}^{i},z_{1:t-1},u_{1:t}\right.)p(s_{t}^{i}\left|z_{1:t-1},u_{1:t}\right.)}{p(s_{t}^{i}\left|z_{1:t-1},u_{1:t}\right.)}\\ =\eta p(z_{t}\left|s_{t}^{i}\right.) \end{array}$$

where η is the normalization coefficient. It can be seen that the weight can evaluate the probability of $z_{t}$ under $s_{t}^{i}$. Normalize the particle weight and update the particle set at time t:

(30) $$w_{t}^{i}=\frac{w_{t}^{i}}{\sum_{i=1}^{N}w_{t}^{i}}$$

The posterior probability density is calculated as follows:

(31) $$s_{t}=\sum_{i=1}^{N}w_{t}^{i}s_{t}^{i}$$

b. Resampling

The effective particle number $N_{eff}$ is expressed as follows:

(32) $$N_{eff}=\frac{1}{\sum_{i=1}^{N}{\left(w_{t}^{i}\right)}^{2}}$$

For the i-th particle, calculate the cumulative sum of particle weights:

(33) $$sum(i)=\sum_{j=1}^{i}w_{t}^{i}$$

Generating random numbers over interval $\partial_{j}∼[0,1]$. These numbers are uniformly distributed.

If $\partial_{j}$ meets the following conditions:

(34) $$sum(j-1)\leq \partial_{j}\leq sum(i-1)$$

Then assign the particle $s_{t}^{i}$ to the j-th particle $s_{t}^{j}$ of the new particle set, and assign the same weight to the particles:

(35) $$w_{t}^{i}=1/N$$

Vouch (2021) [53] et al. proposed a new adaptive unscented particle filter structure that utilizes two cascaded stages to handle disturbed and biased GNSS input observations under harsh conditions. In the upstream stage of redundant measurement noise covariance estimation, a signal processing module based on an inertial navigation system is implemented to enhance the adaptability of observable statistics and improve state estimation. The experimental evaluation of the proposed robust adaptive unscented particle filter algorithm shows that the error is reduced by an average of 10% at more than 75% of the horizontal position of the vehicle. In addition, the proposed method has significantly improved performance compared to ordinary unscented particle filter algorithms.

Geomagnetic matching navigation has the advantages of low cost, wide coverage, and no cumulative error, and is widely used in the positioning and navigation of autonomous robots and vehicles [54]. However, due to the influence of magnetometer measurement noise, the geomagnetic positioning algorithm based on single-point particle filtering may encounter mismatches during continuous operation, which limits its remote positioning performance. To address this issue, Luo (2024) [55] et al. proposed a new real-time geomagnetic localization method based on sequential particle filtering. Firstly, this method reduces the impact of noise during continuous operation while ensuring real-time performance through real-time sequential particle filtering. Then, the trajectory shape of the mileage was corrected using the mileage calibration parameters, which improved the remote positioning accuracy of the method. Finally, the preliminary matching results were subjected to secondary matching using geomagnetic contour matching algorithms, further reducing the localization error of the method. The experimental results show that compared with related algorithms, this method has higher positioning accuracy, with root mean square error, maximum error, and final error reduced by 28.58%, 37.11%, and 0.77%, respectively.

At present, the navigation and positioning accuracy of underwater robots will be affected by the cumulative error of the integrated navigation system, which cannot meet the needs of longer-distance navigation. Based on this, Zhang (2023) [56] et al. proposed to apply the particle filter algorithm to underwater terrain-assisted navigation. Based on the particle filter algorithm, SINS/DVL (strapdown inertial navigation system/Doppler velocity log) integrated navigation information and seabed elevation information are fused. Further, the position information output by the Kalman filter is fused with the particle filter to achieve higher positioning accuracy.

In summary, the particle filter algorithm has the ability to handle nonlinear and non-Gaussian problems, flexibility and generality, multi-sensor fusion capability, real-time performance (within an acceptable range), and suitability for complex dynamic environments in the application of integrated navigation systems. These advantages make the particle filtering algorithm an indispensable and important tool in integrated navigation systems. However, compared to other filters such as Kalman filtering, particle filtering has a greater computational burden and is difficult to meet the high real-time dynamic environment requirements of the system. Especially in complex navigation systems, this issue is particularly prominent. Due to the large computational complexity, particle filtering algorithms perform poorly in terms of real-time performance. In navigation scenarios that require rapid response, this may result in system latency or performance degradation.

At present, there is a lack of a comprehensive indicator system to evaluate the performance of particle filtering algorithms. This makes it difficult to accurately judge the advantages and disadvantages of particle filtering algorithms in practical applications, and to improve and optimize them accordingly.

### 3.5. Neural Network Filtering (NNF)

As the navigation accuracy of INS/GNS will decline with the passage of time [57,58], with the application of machine learning in various fields, the use of artificial neural networks to reduce noise interference in integrated navigation systems and improve positioning accuracy has attracted the attention of many researchers [59,60].

Neural networks learn the inherent patterns of data by constructing neural structures similar to the human brain, enabling machines to have the ability to learn and analyze, thereby achieving the function of predicting data [61]. Commonly used neural networks include fully connected neural networks, convolutional neural networks, recurrent neural networks, etc. Fully connected neural networks are the simplest neural networks, with the most network parameters and the highest computational complexity. The pooling layer of convolutional neural networks will lose a large amount of valuable information and ignore the correlation between local and global aspects. A recurrent neural network is a type of neural network with a feedback structure, which is considered a network structure with a memory function. It has a fast learning ability and high prediction accuracy for input data with time series. However, recurrent neural networks are prone to gradient vanishing, making it difficult to learn information from long distances [62,63].

Doostdar (2019) [64] et al. proposed a heuristic neural network structure based on a recursive fuzzy wavelet neural network to compensate for velocity and position errors in INS, in order to obtain the accuracy of the integrated navigation of GNSS/INS within the inertial navigation gap. Fang (2020) [65] et al. proposed an inertial navigation assistance algorithm based on long short-term memory neural networks to improve navigation accuracy in the event of GNSS failures. The algorithm replaces GNSS signals with long short-term memory neural network algorithms to generate pseudo-GNSS position increments.

Xiao (2021) [66] et al. proposed a confidence estimation algorithm based on a residual attention neural network to address the incompleteness constraints in the integrated navigation of GNSS/INS. The residual attention neural network was used to dynamically estimate the noise covariance of pseudo-observations for optimal Kalman filter fusion. This algorithm introduces an attention mechanism that automatically assigns appropriate weights based on the contribution of learned features. The proposed algorithm was evaluated on three actual road datasets and compared with seven other methods, including traditional optimal Kalman filtering, pure inertial navigation system, deep learning network optimal Kalman filtering, K-means Kalman wave filtering, input delay neural network, etc. Numerous experimental results have shown that the proposed algorithm reduces velocity-related errors and achieves improved accuracy in position and velocity estimation. It can be seen that the auxiliary prediction of integrated navigation through neural networks can greatly improve the accuracy and reliability of integrated navigation.

Musavi (2014) [67] et al. proposed to use fuzzy neural networks to improve the positioning accuracy of integrated navigation systems. The nonlinear system is modeled by a neural network and fuzzy logic method, and the experimental results show that the adaptive fuzzy neural network is superior to the traditional EKF in positioning performance. However, this method can affect the accuracy of positioning when the GPS signal is blocked. Therefore, Zhang (2014) [68] et al. further proposed to combine UKF and BP neural network, which can still provide accurate positioning information even when there is no GPS signal. In addition, M. Malleswaran and Ikram Belhajem proposed dynamic neural network and genetic algorithm neural network algorithms, respectively, to solve the problem of reduced navigation accuracy caused by GPS signal blockage, and verified the effectiveness of the algorithm through experiments [69].

Wang (2015) [70] et al. used a support vector machine regression method to predict the position of vehicles. When the system receives GPS signals, the support vector machine trains a regression model based on GPS and INS data. Therefore, when GPS signals are interfered with, using support vector machine regression models can correct the positioning error of INS and provide positioning accuracy. Aggarwal (2013) [71] et al. proposed a neural network algorithm based on Dempster Shafer theory. Train the Dempster Shafer neural network based on GPS data. When GPS is not available, the trained neural network model can predict the vehicle’s location. The experiment verified the effectiveness of the method. However, neither of these methods can compensate for the prediction error of neural networks.

**Figure 6:**
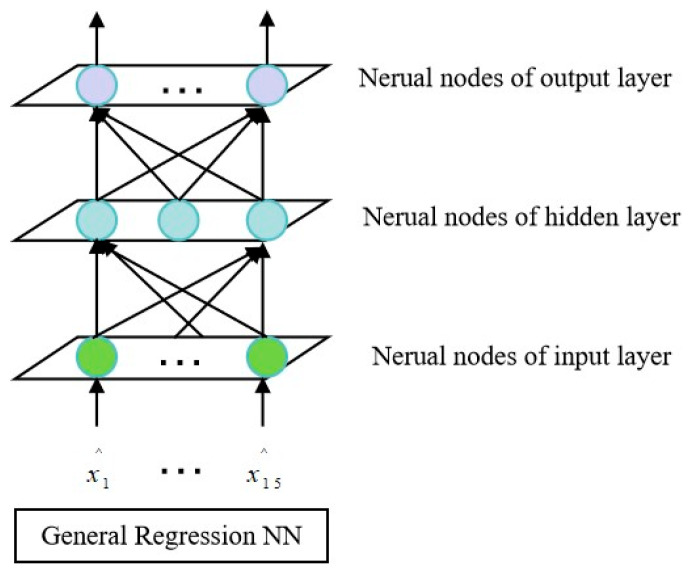
Topology of RBF neural networks.

A radial basis function neural network fusion EKF is proposed for an integrated navigation system [72]. The system combines attitude, heading, and GPS information to provide accurate navigation information for the vehicle. The simulation results show that the fusion filtering algorithm can provide accurate navigation information when the vehicle is in a dynamic environment, even if the measurement accuracy of the sensor is moderate. The algorithm structure is shown in Figure 6.

**Figure 7:**
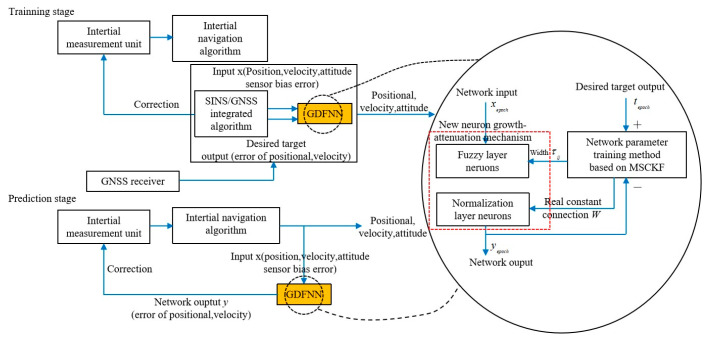
Training stage of the generalized dynamic fuzzy neural network.

The INS/GNS integrated navigation system will seriously affect the navigation accuracy when GNS is interrupted. Therefore, Al Bitar (2021) [73] et al. proposed a navigation algorithm combining UKF and a nonlinear autoregressive neural network, which can predict the position information within the interruption period of GNS. In addition, the prediction accuracy of traditional neural networks largely depends on the network structure and training samples. Therefore, traditional neural networks cannot be directly applied to integrated navigation systems in various environments. Based on this, Wang (2021) [74] et al. proposed a new mechanism of neuron growth and decline based on fuzzy neural network (FNN), which can prevent the network from being over-trained and over-fitted, as shown in Figure 7. At the same time, the strong tracking filter can be introduced to speed up the convergence speed of the neural network and reduce the training time. Simulation experiments show that when GNS signals are lost, the proposed network model reduces latitude and longitude position errors by more than 80% compared to pure inertial navigation methods.

In addition, in order to improve the performance of the integrated navigation system in handling nonlinear signals and uncertain interference, Xiong (2019) [75] et al. proposed a neural network based on a quantum genetic algorithm, which adjusts the gain of KF through the neural network to improve the signal-to-noise ratio and anti-interference ability of the integrated navigation system. The algorithm has been verified to have good accuracy and robustness through simulation tests.

**Figure 8:**
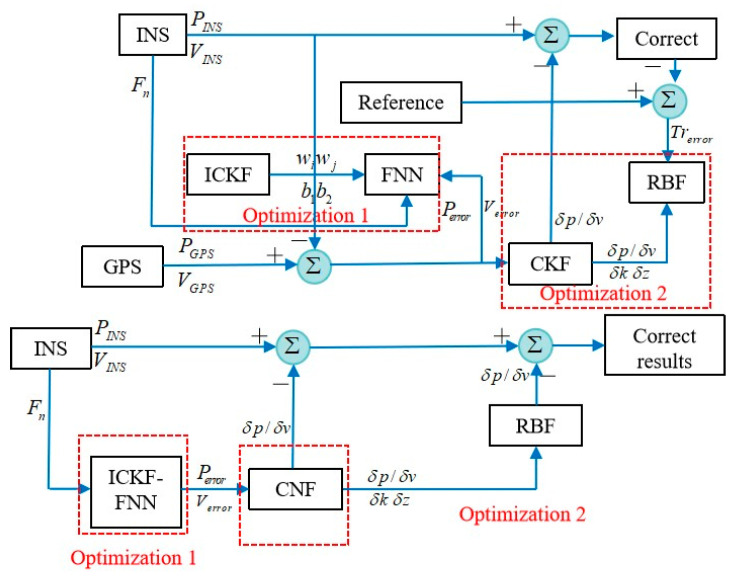
The training model.

At present, there are many studies that combine neural networks with nonlinear filtering methods, but there is relatively little research on how to optimize the parameters of neural networks. Liu (2019) [76] et al. proposed an optimization method using feedforward neural networks and radial basis function neural networks to compensate for errors in the integrated navigation system caused by GPS interruptions. Figure 8 shows the training process of the proposed model. The experimental results show that using the proposed strategy in turning motion reduces the root mean square error of the eastward position by 85.79%, reaching 3.2187 m. The experimental results show that using the proposed strategy in turning motion can reduce the root mean square error of the eastward position by 85.79%. In long linear motion, the cumulative rate of eastward velocity error decreased by 92.69%.

**Figure 9:**
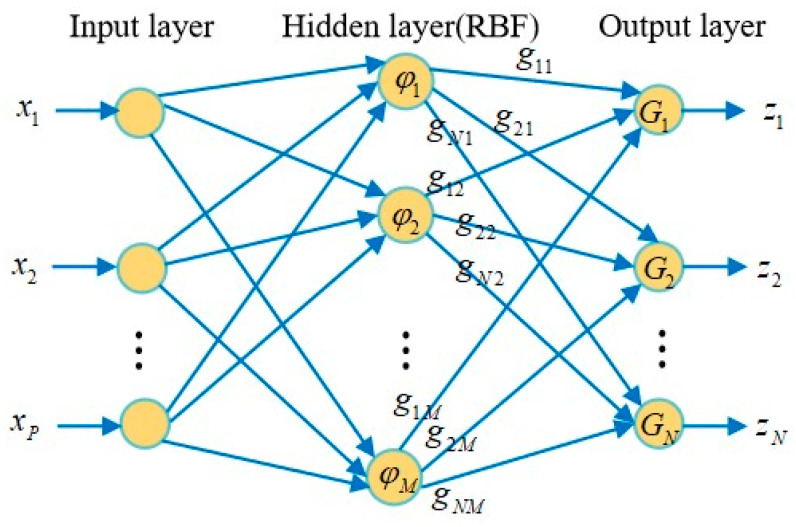
The schematic diagram of the prediction stage.

In addition, Zhao, D. proposed a hybrid pre-estimation method based on an improved UKF, Radial Basis Function Neural Network (RBFNN), and Non-stationary Time Series Analysis (NTSA) [77]. When the system receives GPS signals normally, RBFNN is trained based on the vehicle’s angular velocity, specific force, and navigation error between GPS and SINS. NTSA is trained on the navigation error between the output of RBFNN and GPS and SINS. Then, the navigation error is estimated based on the improved UKF fusion and GPS/SINS data. Further correct navigation errors in the navigation system. In addition, when GPS signals cannot be received, the trained RBFNN and NTSA models can read the vehicle’s position for prediction. The RBFNN structure is shown in Figure 9.

In order to further ensure the good performance of the integrated navigation system in the event of satellite interruption, Yang (2024) [78] et al. proposed a neural network based on strong tracking and UKF to iterate the nonlinear model. At the same time, introducing a strong tracking system to dynamically adjust the gain of the filter can improve the stability and robustness of the neural network, effectively compensate for the accumulated errors of the integrated navigation system, and improve the positioning accuracy.

In summary, neural networks, especially deep learning networks, with their powerful nonlinear fitting and self-learning abilities, can compensate for the shortcomings of Kalman filtering. Although neural networks perform well in integrated navigation, their prediction accuracy and generalization ability still need to be improved. Especially in extreme environments such as high-rise obstruction and electromagnetic interference, the prediction results of neural networks may be greatly affected. In addition, due to the fact that the learning process of neural networks relies on training samples, their prediction results may be biased when the input data undergoes significant changes.

### 3.6. Adaptive/Robust Kalman Filter

#### 3.6.1. Handling Measurement Errors/Outliers/Faults

The complex environment requires the integrated navigation system to have sufficient adaptability to cope with time-varying measurement noise, while also requiring the system to have sufficient robustness to cope with abnormal measurement values and system prior uncertainty. However, low-cost GNSS/INS integrated navigation systems are difficult to simultaneously balance the above two performance aspects.

Due to the degradation of GNSS signals in harsh environments such as valleys, tunnels, and forests, INS/GNSS integrated systems inevitably experience measurement outliers, leading to a decline in the performance of the integrated navigation system. In practical applications, the statistical characteristics of measurement noise are unknown and time-varying, and are prone to sudden changes, leading to deviations or even divergence in KF solutions. Therefore, in order to improve the robustness of KF, it is necessary to study the statistical characteristics of time-varying measurement noise. Gao (2025) [79] et al. proposed a new robust Kalman filter that applies kernel density estimation to the Kalman filter to address the interference of measurement outliers on system state estimation. Simulation and experimental results show that the proposed RKF outperforms KF and PF in state estimation of vehicle-integrated navigation systems in the presence of measurement outliers.

**Figure 10:**
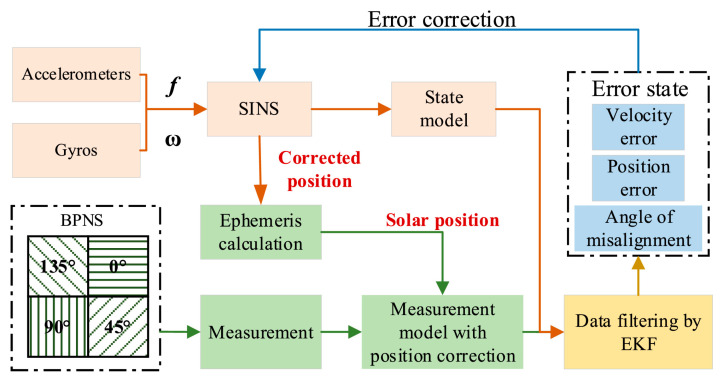
A tightly coupled SINS/BPNS system with position correction.

In order to correct the accumulated errors of the strapdown inertial navigation system for autonomous navigation of unmanned aerial vehicles, SINS/BPNS (Bionic po linearization navigation system) integrated navigation has been proven to be a promising navigation strategy to replace SINS/GNSS in environments where GNSS cannot be used. Gao (2024) [80] et al. proposed an enhanced tightly coupled SINS/BPNS integrated navigation system based on focal plane polarization sensors, which effectively corrected the position of SINS in the SINS/BPNS combination. The SINS/BPNS system is shown in Figure 10. Further combined with extended Kalman filtering, the filtering robustness of the tightly coupled SINS/BPNS combination has been improved. Simulation and experimental results show that compared with tightly coupled integration with position correction, the latitude and longitude positioning accuracy are improved by at least 3.02% and 10.82%, respectively.

**Figure 11:**
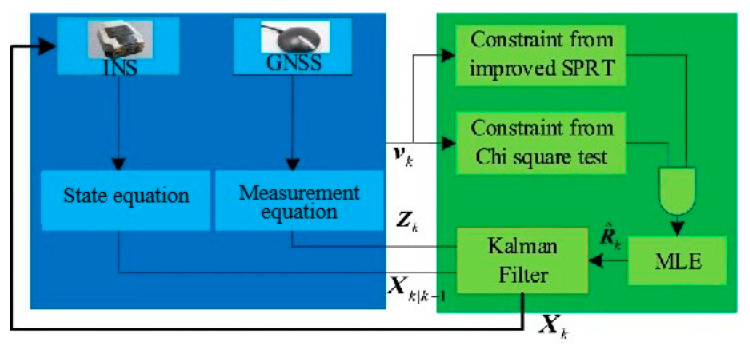
The framework of the robust Kalman filter algorithm is based on hypothesis testing constraints.

In addition, Gao (2023) [81] et al. proposed a new method for INS/GNSS integrated navigation in the presence of measurement anomalies, shown in Figure 11. This method combines hypothesis testing with maximum likelihood theory to estimate the measurement noise covariance of measurement outliers. A robust Kalman filtering algorithm for tightly coupled INS/GNSS integrated navigation is proposed based on hypothesis testing, constrained maximum likelihood estimation of observed noise covariance. Simulation and experimental results, as well as comparative analysis, show that this method can effectively handle abnormal measurements, with accuracies 46% and 30% higher than KF and Maximum Likelihood-Robust KF, respectively. Gao (2019) [82] et al. combined CKF with the Mahalanobis distance criterion and proposed a new robust Kalman filtering algorithm with a scaling factor. Introduce a robustness factor calculated using the Mahalanobis distance criterion in the standard CKF to magnify the covariance of measurement noise and reduce filtering gain in the presence of anomalous observations. The proposed robust CKF can effectively resist the impact of abnormal observations on navigation performance, thereby improving the robustness of CKF in vehicle INS/GNSS integration.

Hu (2019) [83] et al. studied the nonlinear state estimation problem in the navigation of hypersonic aircraft. Due to the high maneuverability of hypersonic aircraft, tightly coupled INS/GNSS inevitably suffers from measurement errors, such as outliers in pseudorange observations and non-Gaussian noise distributions. A robust unscented Kalman filter based on orthogonality was proposed to suppress the interference of measurement errors on navigation performance. Based on hypothesis testing theory, measure measurement errors and further adjust the Kalman gain to reduce the weight of error measurement, ensuring filtering robustness against measurement errors. The proposed robust unscented Kalman filter avoids the decrease in state estimation accuracy without measurement errors.

In summary, various robust KF algorithms can handle outliers and non-Gaussian measurement noise in nonlinear systems in INS/GNSS integrated navigation systems. By combining kernel density estimation, hypothesis testing, and maximum likelihood theory, as well as robust UKF, the robustness of the system can be improved through online estimation of noise statistics. However, in practical applications, these methods may encounter the problem of “insufficient rank” in the calculation of noise statistics for high-dimensional systems, leading to stability issues. Secondly, the estimation of noise statistics based on historical residuals has a lag, making it difficult to adapt to highly dynamic environments. For the improvement direction of outlier detection, a deep learning-based outlier recognition module can be used, such as convolutional neural networks for spatiotemporal feature extraction of GNSS residual sequences, combined with long short-term memory networks to achieve dynamic classification of outliers. The adaptive noise covariance adjustment strategy enhances the robustness of the system to sudden outliers by dynamically adjusting the filtering gain through online estimation of measurement noise variance.

#### 3.6.2. Handling System Model Error/Uncertainty

The INS/CNS integrated navigation system has characteristics such as strong nonlinearity, non-additive noise, and dynamic complexity. The INS/GNSS integrated navigation system is mainly affected by inherent kinematic model errors. The existing research on tightly coupled INS/CNS combinations is mainly based on linear systems obtained from the first-order Taylor expansion of actual nonlinear systems. In order to reduce computational costs, the dynamic model is linearized, assuming that the initial state estimation contains small errors. However, in most practical applications, it is difficult to satisfy this assumption, especially for targets with high dynamic characteristics, as the true initial state is unknown. Therefore, traditional KF or EKF have poor navigation accuracy for high dynamic targets in tightly coupled INS/CNS combinations.

**Figure 12:**
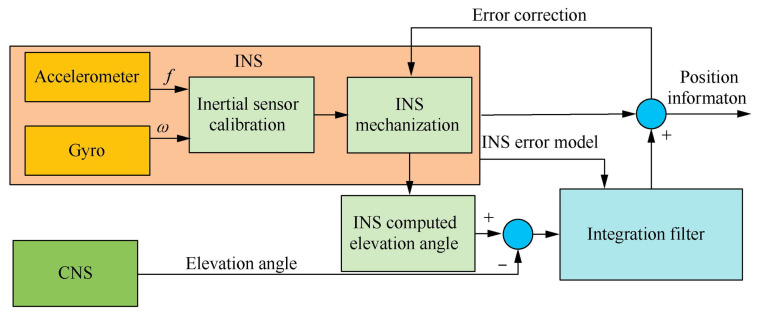
The schematic diagram of a tightly coupled INS/CNS.

To address the issues in tightly coupled INS/CNS integrated navigation, Gao (2022) [84] et al. proposed introducing state augmentation techniques into traditional augmented state functions to reduce the negative impact of non-additive noise in dynamic models. The schematic diagram of Tightly coupled INS/CNS is shown in Figure 12. A detection method for dynamic model uncertainty has been established, and a fading factor has been rigorously derived based on Mahalanobis distance theory, thereby improving the augmented CKF to handle the uncertainty of dynamic models. The simulation results and comparative analysis show that this method can effectively suppress the adverse effects of non-additive noise and dynamic model uncertainty on inertial measurement, resulting in higher navigation accuracy than traditional EKF and CKF.

For the INS/GNSS combination system of hypersonic aircraft, due to its complex flight dynamics and high maneuverability, it is impossible to accurately establish the aircraft dynamics model. Therefore, the navigation performance of hypersonic aircraft is mainly affected by the inherent errors of the dynamic model, and it is required that the navigation parameter analysis nonlinear filtering algorithm used has the ability to handle dynamic model errors. In order to improve the estimation performance of UKF in the presence of dynamic model errors, a model predictive-based unscented Kalman filter (MP-UKF) algorithm was proposed [85]. MP-UKF utilizes the concept of model predictive filtering to establish an estimator of dynamic model errors, and then compensates for the model error estimation in the UKF process used for nonlinear state estimation. The proposed MP-UKF can persistently correct dynamic model errors caused by UKF, thus overcoming the limitations of UKF.

Aiming at the motion model error of the integrated navigation system, Hu (2018) [86] et al. combined the inertial navigation equation and the inertial measurement unit error equation to establish the kinematics model of the INS/GNSS integrated navigation system. Further, a refined strong tracking unscented Kalman filter (RSTUKF) was proposed to enhance the robustness of UKF to kinematics model errors. This RSTUKF adopts the strategy of hypothesis testing to identify kinematics model errors. On this basis, the suboptimal fading factor is derived and embedded in the prediction covariance to attenuate the influence of the prior information on the filtered solution only in the presence of kinematic model errors. In addition to correcting the UKF estimate in the presence of kinematic model errors, RSTUKF also maintains the optimal UKF estimate in the absence of kinematic model errors. Simulation and experimental results show that the filter can effectively resist the interference of motion model errors on INS/GNSS state estimation, and has higher navigation accuracy than UKF and STUKF.

In order to solve the nonlinear problem in INS/GNSS integrated navigation, an adaptive UKF with noise statistical estimator was proposed [87]. Based on the covariance matching technique, the covariance matrix of process noise and measurement noise is determined using the innovative sequence and the residual sequence. This algorithm can estimate and adjust system noise statistics online, thereby enhancing the adaptive capability of the standard UKF. Simulation and experimental results show that without an accurate understanding of system noise, the performance of this algorithm is significantly better than that of standard UKF and adaptive robust UKF, thereby improving navigation accuracy.

Gao (2015) [88] et al. proposed a random weighting method to estimate the error characteristics of INS/GPS/SAR integrated navigation systems. An error model was established to represent and analyze white noise errors in INS/GPS/SAR integrated navigation systems. Estimate the statistical characteristics of white noise errors based on error models and random weighting theory. The experimental results show that the proposed random weighting method can effectively estimate the white noise error in the INS/GPS/SAR integrated navigation system, and the accuracy is much higher than that of the least squares method.

**Figure 13:**
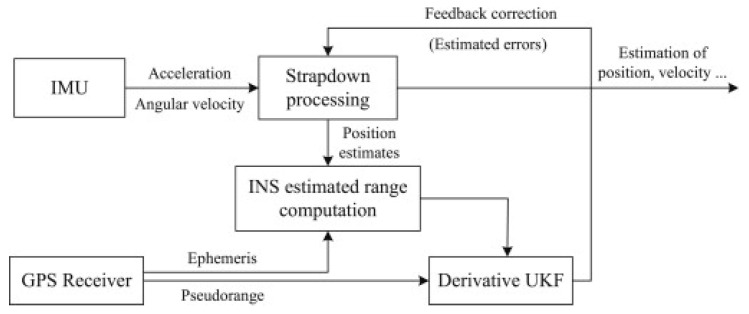
The schematic diagram of the tightly coupled GPS/INS based on the derivative UKF.

The tightly coupled INS/GPS combination, due to the use of raw GPS pseudorange measurements, results in a nonlinear measurement equation for the Kalman filter. EKF is a typical method for handling nonlinearity by linearizing pseudo-range observations. However, linearization may lead to significant modeling errors and even degradation of navigation solutions. To address this issue, Hu (2015) [89] et al. constructed a nonlinear measurement equation by adding a second-order term to the Taylor series of pseudorange observations, shown in Figure 13. However, when applying UKF to INS/GPS integrated navigation estimation, the linear nature of the system state equation can result in a significant number of redundant calculations during the prediction process, especially when the system state vector is higher-dimensional than the measurement vector. To overcome this computational burden limitation, a derivative UKF was further developed based on the constructed nonlinear measurement equation. The derivative UKF is predicted using the simplified form of the original KF and updated using the traceless transformation technique. Theoretical analysis and simulation results indicate that the derivative UKF has higher accuracy and smaller computational complexity compared to the traditional UKF.

KF requires that the kinematic and observational models not contain any systematic errors. Otherwise, the obtained navigation solution will have deviation or even divergence. To overcome this limitation, Wei (2016) [90] et al. proposed a random weighting method to estimate the systematic error of the observation model in dynamic vehicle navigation. This method randomly weights the covariance matrices of the observed residual vector, predicted residual vector, and estimated state vector to control their sizes, thereby controlling the random weighted estimation of the covariance matrix of the observed vector. We established the theory of random weighting to estimate the systematic error of the observation model, as well as the covariance matrix of the observation residual vector, prediction residual vector, observation vector, and estimated state vector. Experiments and comparative analysis with existing methods show that the proposed random weighting method can effectively resist the interference of observation model system errors on state parameter estimation, thereby improving the accuracy of dynamic vehicle navigation.

In addition, Sage windowing and random weighted adaptive filtering methods have also been proposed to estimate kinematic model errors in aircraft navigation [91]. This method dynamically adjusts the covariance matrix of the predicted residual vector and state noise vector through random weights to control the covariance matrix of the predicted state vector, thereby reducing the interference of kinematic model errors on system state estimation.

#### 3.6.3. Handing Both Model and Measurement Uncertainties

**Figure 14:**
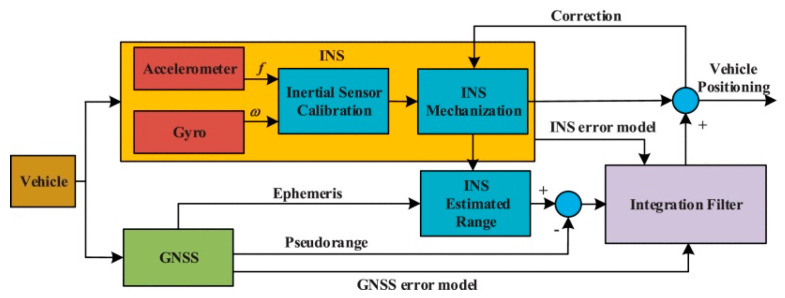
A tightly coupled GNSS/INS integrated framework for vehicle positioning.

When tightly coupled GNSS/INS integrated navigation systems are simultaneously affected by system model errors and measurement noise uncertainties, the navigation accuracy will be severely reduced. Gao (2021) [92] et al. proposed an adaptive and robust CKF based on Mahalanobis distance for the tightly coupled GNSS/INS combination positioning problem with system modeling errors. The schematic diagram of the tightly coupled GPS/INS-based derivative UKF is shown in Figure 14. A motion modeling error fading memory adaptive CKF based on the Mahalanobis distance was derived without involving human experience. On this basis, a new CKF with both adaptability and robustness was established by integrating the results of standard CKF, adaptive CKF, and robust CKF using the IMM principle. Simulation and experimental results, as well as comparative analysis, indicate that the proposed methodology can effectively suppress the interference of kinematic and observational modeling errors on the state.

UKF has become a promising method for state estimation of nonlinear dynamic systems due to its simple calculation process and superior performance in highly nonlinear systems. However, when there is uncertainty in the system model, its solutions will degrade or even diverge. Gao (2017) [93] et al. proposed an adaptive robust UKF based on Interactive Multi Model (IMM) estimation to address this issue. This method combines the advantages of adaptive fading UKF and robust UKF, and suppresses the interference of system model uncertainty on the filtered solution. Based on the principle of orthogonality, adaptive fading UKF for process model uncertainty and robust UKF for measurement model uncertainty were established. Subsequently, an interactive multi-model estimation was developed, which fused adaptive fading UKF and robust UKF into sub-filters. System state estimation is achieved by weighting the probabilities of the estimated results from two sub-filters. The effectiveness of this method has been verified through simulation and experiments.

The SINS/SAR (Strapdown Inertial Navigation System/Synthetic Aperture Radar) integrated navigation system utilizes relevant images to obtain positioning information.

However, the state model of the SINS/SAR integrated navigation system has the characteristics of low process noise and exponential instability. The errors caused by the instability of the system state model accumulate continuously during the navigation process, reducing the accuracy of navigation. In addition, due to the instability of the system and the uncertainty of the environment, the statistical characteristics of the system state and observation noise may not be accurately described, leading to inaccuracies in the SINS/SAR integrated navigation system. Therefore, Gao (2011) [94] et al. proposed a robust adaptive filtering method for SINS/SAR (Strapdown Inertial Navigation System/Synthetic Aperture Radar) integrated navigation system. This method adopts the principle of robust estimation to adaptively filter the observed data. A robust adaptive filter has been developed to adaptively determine the covariance matrix of observation noise and adaptively adjust the covariance matrix of system state noise based on an adaptive factor constructed from prediction residuals. The experimental results and comparative analysis show that this method can not only effectively resist the interference caused by system state noise and observation noise, but also achieve higher accuracy than the adaptive Kalman filtering method.

In addition, Gao (2011) [95] et al. proposed a dynamic navigation positioning random weight estimation method to reduce the accuracy of navigation estimation due to errors in kinematics and observation models. This method adopts the concept of random weighted estimation to estimate the covariance matrix of system state noise and observation noise, in order to control the interference of singular observations and kinematic model errors. Satisfy the practical requirements of fully utilizing observation information through residual vectors and innovation vectors, thereby reducing the interference of kinematic model errors and observation model errors on state parameter estimation. This random weight estimation method provides an effective solution for improving positioning accuracy in dynamic navigation.

The existing robust adaptive filtering methods mainly focus on combining robust estimation theory in the adaptive filtering process, without considering the system model errors in the adaptive filtering process. In addition, these methods mainly focus on integrated navigation systems, and research on using robust adaptive filtering for transmission alignment is very limited. Gao (2016) [96] et al. proposed a new robust adaptive filtering method that considers the systematic errors of observation and kinematic models during the filtering process for transfer alignment. This method overcomes the limitations of traditional Kalman filters, which require precise kinematic and observation models for transfer alignment. It adaptively adjusts and updates prior information through an equivalent weight matrix and adaptive factors to resist the interference of system model errors on system state estimation, thereby improving the accuracy of state parameter estimation. The experimental results and comparative analysis show that the proposed robust adaptive filtering method can effectively improve the performance of transmission alignment, and the achieved performance is much higher than that of the Kalman filter and the traditional robust adaptive Kalman filter.

#### 3.6.4. Handling Uncertainties Caused by Unknown/Inaccurate Process and Measurement Noise Covariances

The Kalman filter is an important technique for system state estimation. It requires accurate knowledge of system noise statistics to achieve optimal state estimation. However, in practice, due to the uncertainty and interference involved in dynamic environments, this knowledge is often unknown or inaccurate, leading to the degradation or even divergence of Kalman filtering solutions.

The existing research on the interference of inaccurate noise statistics on state estimation is mostly limited by Kalman or Bayesian frameworks, and still requires more or less knowledge of system noise statistics, resulting in a limited ability to handle noise statistical interference. The H∞ filter performs system state estimation by minimizing the peak estimation error rather than the average error variance in KF. Its state estimation does not require any system noise statistical information; therefore, it has strong anti-interference ability for unknown noise statistics. Therefore, the system state information generated by the H∞ filter provides a reliable source for noise statistical estimation.

Gao (2025) [97] et al. proposed a new H∞ filtering method based on adaptive random weighting estimation. It combines H∞ filters with the concept of random weighting to estimate system noise statistics. Establish a random weighting theory based on an H∞ filter for state estimation and state error covariance to estimate process noise statistics and measurement noise statistics. Subsequently, the estimated system noise statistics are fed back into the Kalman filtering process for system state estimation. Simulation and experimental results show that this method can effectively estimate the system noise statistics, thereby improving the accuracy of system state estimation.

Random weighted estimation is an advanced statistical calculation method. This method applies random weights to estimate target parameters for different samples. It can achieve unbiased estimation, with simple calculations, and can also handle large samples without the need for precise distribution of target parameters. Gao (2024) [98] et al. proposed combining the concept of random weighting with finite memory techniques to accurately estimate system noise statistics. In order to avoid the influence of excessive historical information on state estimation, a random weighting theory is established based on limited memory technology to estimate process noise and measurement noise statistics within a limited memory. Subsequently, the estimated system noise statistics are fed back into the Kalman filtering process for system state estimation. This method improves the accuracy of Kalman filtering by adaptively adjusting the weight of system noise statistics within a limited memory, and suppresses the interference of system noise on system state estimation.

With the completion of China’s Beidou-3 system (BDS), INS/BDS fusion will become a promising navigation and positioning strategy. However, due to the nonlinear propagation characteristics of INS errors and the inevitable involvement of inaccurate measurement noise statistics, it is difficult to achieve optimal solutions through INS/BDS fusion. Gao (2021) [99] et al. proposed a CKF method that utilizes the maximum likelihood principle for estimating the covariance of measurement noise to address the aforementioned issues. Establish a measurement noise covariance estimation model based on the maximum likelihood principle, and then use sequential quadratic programming to calculate its estimation. The estimated measurement noise covariance will be fed back to the CKF program to improve its adaptability. Simulation analysis has demonstrated that this method can accurately estimate the covariance of measurement noise, effectively suppress its impact on navigation solutions, and thus improve the navigation performance of INS/BDS integration.

In addition, when applying UKF to INS/GPS integrated navigation systems, it highly relies on prior statistical information of process and measurement noise. If inaccurate noise statistics are used, UKF performance will decrease. The concept of Maximum Likelihood has been applied to adaptive filtering. However, all existing filtering methods based on Maximum likelihood are derived within the framework of linear systems and are not suitable for nonlinear state estimation. Due to the difficulty of theoretical derivation, research on using Maximum Likelihood estimation to improve UKF performance is very limited in the presence of inaccurate noise covariance in nonlinear state estimation.

Hu (2020) [100] et al. proposed a new adaptive UKF with process noise covariance estimation based on the Maximum Likelihood principle to enhance the robustness of UKF to process noise uncertainty. This method extends the concept of Maximum Likelihood estimation from the linear Kalman filter to the nonlinear UKF for estimating process noise covariance. Due to its ability to estimate and update process noise covariance online, this method improves the standard UKF by suppressing the interference of process noise uncertainty on the filtered solution.

Gao (2020) [101] et al. proposed a new adaptive UKF based on the concepts of maximum a posteriori and random weighting. Calculate the noise statistic based on the principle of maximum a posteriori probability, and then use the concept of random weighting to optimize the maximum a posteriori estimate obtained by adjusting the weights of residuals online. We have established maximum a posteriori estimation and random weighted estimation of noise statistics for online estimation and adjustment of system noise statistics, thereby improving the robustness of filtering.

For strongly nonlinear and Gaussian systems, CKF outperforms UKF in terms of accuracy and stability. Zong (2019) [102] et al. proposed incorporating random weighted estimation into CKF to improve filtering performance. This method directly modifies the weight of volume points based on prediction errors, rather than estimating system noise statistics, to suppress the impact of system errors on system state estimation. It establishes the theory of random weighting to estimate the predicted system state, system measurements, and their error covariance. Based on these theories, the proposed random weighted CKF dynamically modifies the weight of volume points according to prediction errors to suppress the interference of system errors on system state estimation and improve filtering accuracy.

#### 3.6.5. Chapter Summary

In engineering practice, due to the uncertainty and disturbance in dynamic environments, the statistical characteristics of system noise are often unknown or inaccurate, leading to deviations or even divergence in KF solutions. Adaptive filtering suppresses the impact of inaccurate process noise statistics on system state estimation by introducing adaptive factors in KF, while robust filtering suppresses the impact of inaccurate measurement noise statistics on system state estimation by introducing robust factors in KF. However, these two methods cannot guarantee accuracy as the determination of adaptive and robust factors relies on experience. The maximum likelihood principle estimates the system noise statistic by maximizing the joint likelihood function related to the system state and measurement. However, due to the equal contribution of historical residuals to the evaluation of system noise statistics, this method cannot accurately reflect the statistical characteristics of system noise, resulting in a decrease in filtering accuracy.

Covariance matching is a technique for updating the system noise covariance by matching the actual residual covariance with its theoretical value. However, this technology involves the calculation of partial derivatives, resulting in an increase in computational complexity. Fuzzy logic can detect system noise/uncertainty to improve the adaptability and robustness of KF. However, fuzzy rules are developed based on empirical and heuristic information, resulting in limited performance. Sage Husa adaptive filtering is a technique for handling system model errors or unknown noise statistics to prevent a decrease in KF accuracy or even divergence [24]. However, since the forgetting factor used in this filter is determined based on experience, it may not be guaranteed that the resulting solution is optimal. Cui and Niu also studied neural network methods in the KF framework to match unknown system errors/noise to improve performance. But this method relies on a large number of samples to ensure accuracy.

Random weighted estimation is a statistical calculation method. It can provide unbiased estimation, is computationally simple, and is suitable for large samples even with unbounded noise statistics. Unlike maximum likelihood estimation, random weighted estimation uses different weights to characterize the contribution of each sample to parameter estimation, thereby improving estimation accuracy. However, the estimation accuracy of random weighting methods still depends on sample information. Overall, existing research on the interference of inaccurate noise statistics on state estimation is mostly limited by Kalman or Bayesian frameworks, and still requires more or less knowledge of system noise statistics, resulting in limited ability to handle noise statistical interference.

Due to the ability of H∞ filters to provide reliable system state information regardless of system noise information, incorporating random weighting methods into the H∞ filter framework is a promising solution for statistical estimation of system noise. This method can effectively estimate the system noise statistics, thereby improving the accuracy of state estimation.

## 4. Discussion

As one of the earliest nonlinear filtering algorithms, EKF has been widely used in integrated navigation systems. Linearizing and approximating nonlinear functions through Taylor expansion makes the algorithm implementation relatively simple. The existing EKF research methods focus on reducing the impact of noise covariance uncertainty. However, when the nonlinearity of the system is strong, the first-order linearization approximation of the Taylor expansion may introduce significant errors, affecting the filtering accuracy. The need to calculate the Jacobian matrix of nonlinear functions increases the computational complexity of the algorithm [103,104,105].

Optimize the linearization process in the EKF algorithm to reduce the impact of linearization errors on state estimation. For example, higher-order Taylor series can be used to approximate nonlinear functions, or adaptive linearization techniques can be introduced to dynamically adjust linearization parameters. Strengthen the analysis and processing capabilities of system noise and observation noise, and adopt more reasonable noise models to describe noise characteristics. Meanwhile, noise suppression and filtering techniques are introduced to reduce the impact of noise on state estimation. Optimize the calculation process of the EKF algorithm to improve computational efficiency for large-scale data processing and high real-time requirements. For example, parallel computing technology can be used to accelerate the execution speed of algorithms; Alternatively, the computational complexity can be reduced by simplifying the algorithm structure and minimizing unnecessary calculation steps. The application of the extended Kalman filter algorithm in integrated navigation will continue to develop towards high accuracy, robustness, multi-source data fusion, intelligence and autonomy, as well as low power consumption and miniaturization in the future. Meanwhile, by optimizing the system model, improving linearization methods, introducing adaptive filtering techniques, enhancing noise processing capabilities, and improving computational efficiency, the performance and application effectiveness of the EKF algorithm in integrated navigation can be further improved [106,107,108].

UKF has higher nonlinear approximation accuracy than EKF and does not require the calculation of the Jacobian matrix. By using the traceless transformation to handle the mean and covariance transfer of nonlinear functions, the errors caused by linearization approximation are avoided, and the filtering accuracy is improved. The performance of the UKF algorithm largely depends on the selection of sampling points. Inappropriate sampling points may lead to a decrease in filtering accuracy and even cause filtering divergence. In addition, the UKF algorithm also assumes that the system noise and observation noise are Gaussian, but in practical applications, this assumption may not hold true. The inaccuracy of noise statistical characteristics can affect the stability and accuracy of filters. Therefore, for complex nonlinear systems, the nonlinear processing capability of the UKF algorithm can be further improved. For example, combining other nonlinear filtering methods, such as particle filtering for hybrid filtering, or introducing more advanced nonlinear approximation techniques to improve filtering accuracy [109,110].

The CKF algorithm overcomes the limitations of the Unscented Kalman Filter algorithm and the Extended Kalman Filter algorithm, and has better stability. However, when dealing with high-dimensional nonlinear systems, the CKF algorithm requires the calculation of multiple volume points and their corresponding weights, which is computationally intensive. Real-time performance decreases, and system response speed slows down. Future research can further explore how to dynamically adjust the system model and noise covariance matrix based on real-time data to better adapt to changes in the actual navigation environment. This can be achieved by introducing adaptive forgetting factors, sliding windows, and other methods. Optimize the number and location of volume points to reduce computational complexity and improve estimation accuracy. For example, sparse volume point sets can be used, or the distribution of volume points can be adjusted based on system characteristics.

In integrated navigation systems, traditional filtering algorithms such as Kalman filtering and its extended forms often struggle to achieve ideal accuracy and robustness due to the nonlinear and non-Gaussian characteristics of sensor data. Particle filtering algorithm, as a Monte Carlo method based on Bayesian theory, approximates the posterior probability distribution of the system through a set of weighted particles, which can effectively handle nonlinear and non-Gaussian problems. Therefore, it has been widely used in integrated navigation. The particle filter algorithm requires an increase in the number of posterior probability density samples to be described when dealing with high-dimensional system models, resulting in a significant increase in computational complexity. Compared to other filters, such as the Kalman filter, the particle filter has a greater computational burden and is difficult to meet the high real-time dynamic environment requirements of the system. Due to the large computational complexity, particle filtering algorithms perform poorly in terms of real-time performance. In navigation scenarios that require rapid response, this may result in system latency or performance degradation. Future research will focus on developing more efficient particle count optimization methods to balance computational complexity and filtering accuracy. In addition, the number of particles is dynamically adjusted based on real-time changes in system status and observation data to reduce computational costs while maintaining filtering accuracy.

Neural network algorithms can discover patterns and rules in sensor data through data training and learning, enabling more accurate data classification and decision-making, and improving the positioning accuracy and stability of integrated navigation systems. Especially in complex environments where GPS signals are unavailable or interfered with, neural network algorithms can predict and correct navigation parameters by learning data from other sensors, ensuring the continuity and reliability of the system. However, when satellite signals are interrupted, the prediction accuracy of existing neural networks is not high, and their generalization ability is poor. This is mainly because the learning algorithm of neural networks relies on training samples and has low training efficiency, making it difficult to cope with complex and changing navigation environments. Further optimization of the structure and algorithms of neural networks is needed to improve their prediction accuracy and generalization ability in complex environments. In addition, the training process of neural networks requires a large amount of computing resources and time, and has high requirements for the quality and quantity of training samples. In practical applications, how to quickly and efficiently train neural networks while reducing their dependence on training samples is an urgent problem to be solved.

Future neural network algorithms will pay more attention to the fusion processing of multi-sensor data. Integrated navigation systems typically include multiple sensors (such as GPS, INS, visual sensors, etc.) that provide different types of data, but all play an important role in improving navigation accuracy. Neural network algorithms will be able to more effectively integrate these multimodal data, achieving more comprehensive environmental perception and more accurate navigation positioning. Additionally, further research is required. How to maintain stable performance output of neural networks under adverse conditions, such as noise interference and signal loss. Due to the advantages and disadvantages of each filtering algorithm, how to organically combine EKF, UKF, CKF, PF, and NNF to design various adaptive filtering algorithms based on interactive multi-modeling to solve the dynamic filtering problem of nonlinear systems in integrated navigation [111,112,113]. The future development direction of adaptive filtering and robust filtering in INS/GNSS integrated navigation systems includes the integration with intelligent technologies such as deep learning to improve the modeling ability of nonlinear errors; Develop more efficient adaptive noise compensation technology and intelligent fusion algorithm; Enhance anti-interference and fault tolerance to adapt to a complex and changeable application environment. The comparison analysis of nonlinear filtering algorithms (EKF, UKF, CKF, PF, NNF, AKF, and Robust KF) is shown in Table 1.

## 5. Conclusions

This manuscript introduces the research status of nonlinear filtering algorithms in integrated navigation systems, and explores the principles and applications of EKF, UKF, CKF, PF, NNF, and Adaptive/Robust KF in integrated navigation. Compared to other nonlinear filtering algorithms, the implementation of EKF is relatively simple and easy to implement in engineering. EKF requires calculating the Jacobian matrix of nonlinear functions, which increases the complexity of the calculation. UKF is suitable for various nonlinear systems, especially strongly nonlinear systems, and can provide more accurate state estimation. UKF approximates the probability distribution of the system through the Unscented Transform, avoiding linearization errors in EKF and thus having higher filtering accuracy. PF is applicable to any nonlinear non-Gaussian system that can be represented by a state space model and has strong flexibility. The computational complexity of PF is usually large, and particle degradation issues can also affect its filtering performance. In addition, through deep neural networks, complex signals and noise can be more accurately modeled, thereby achieving more effective filtering. In the future, the technology of neural network-assisted integrated navigation will continue to develop, and the accuracy and reliability of integrated navigation will be greatly improved.

## Figures and Tables

**Table 1:** Comparative analysis of nonlinear filtering algorithms.

Filter Type	Advantages	Disadvantages	Computational Complexity	Accuracy
KF	Linear system is optimal under Gaussian noise; Computational efficiency.	Linear systems only; Performance degradation in nonlinear/non-Gaussian scenes.	Minimum	High (linear Gaussian scene)
EKF	Extended to weakly nonlinear systems; Simple implementation.	Linearization error; Taylor expansion approximation error; Computational burden of Jacobian matrix.	low	Medium (weakly nonlinear scenario)
UKF	Avoid linearization error; High precision of strong nonlinear system; No Jacobian matrix required	The calculation amount is greater than EKF; Parameter selection sensitivity.	Moderate	High (strongly nonlinear scenario)
CKF	Numerical stability is generally better than UKF; The accuracy is equivalent to UKF.	High computational complexity of high-dimensional system.	Moderate	High (low dimesional)
PF	Dealing with arbitrary nonlinear/non-Gaussian systems; High flexibility.	High calculation cost; Particle degradation problem; Low efficiency of high-dimensional state space.	Highest	High (non-Gaussian/non-linear scene)
NNF	Strong nonlinear mapping ability; Adaptive learning of complex relationships.	Requires a large amount of training data; Particle degradation; Complex implementation (requires resampling).	Offline training: extremely high Online reasoning: medium high	High (when fully trained)
AKF	Dynamic noise adjustment to adapt to time-varying environment, strong nonlinear processing	Parameter sensitivity, easy divergence and high computational complexity	High	High
Robust KF	High fault tolerance, stable accuracy	Non-Gaussian scenes require additional design and iteration costs	Medium (high dimension needs to be weighed)	High
